# Gastric Mucormycosis in a Critically Ill Patient Who Survived Without Surgery: A Case Report

**DOI:** 10.7759/cureus.86140

**Published:** 2025-06-16

**Authors:** Andrej M Sodoma, Atieh D Ashkezari, James R Pellegrini, Shweta Chaudhary, Shaliesha Hinds Allen

**Affiliations:** 1 Internal Medicine, South Shore University Hospital, Bay Shore, USA; 2 Internal Medicine, New York Institute of Technology College of Osteopathic Medicine, Old Westbury, USA; 3 Internal Medicine, Nassau University Medical Center, East Meadow, USA; 4 Pathology, Northwell Health, Bay Shore, USA; 5 Gastroenterology, Northwell Health, Bay Shore, USA

**Keywords:** acute gi bleed, diabetic ketoacidosis, immunocompromised patient, invasive fungal infections, uncontrolled diabetes

## Abstract

Gastrointestinal mucormycosis is an extremely rare but highly fatal manifestation of invasive fungal infections. We report a case of a 57-year-old man with preexisting type 2 diabetes mellitus who was admitted for diabetic ketoacidosis and acute hypoxic respiratory failure with a stay in the intensive care unit. The patient was found to be anemic. On further workup, he was found to have gastric mucormycosis. Despite the very high mortality rate, the patient showed remarkable improvement with antifungal therapy and supportive care. No surgery was performed as the patient refused surgical intervention. This rare case points to the need for high suspicion, timely biopsy, and quick intervention to have favorable outcomes.

## Introduction

Mucormycosis is a rare but severe fungal infection caused by a group of fungi called Mucorales. It is commonly seen in diabetic patients due to a weakened immune system, especially when they are in diabetic ketoacidosis (DKA). In this state of stress, the body releases iron into the bloodstream, allowing fungi to consume it and grow [1]. The incidence of mucormycosis is between 0.005 and 1.7 cases per million population per year [2]. It most commonly occurs in organ transplant patients and diabetic patients. It accounts for approximately 2% of all invasive fungal infections in patients undergoing solid organ transplants [3]. In the United States, in 2019, there were 1,140 hospitalizations associated with mucormycosis [3]. The mortality rate of mucormycosis is roughly 50%. Mucormycosis can occur in various locations, including rhino-orbito-cerebral mucormycosis, the most common overall involvement. The lungs, skin, and the gastrointestinal (GI) tract can also be involved. The least common location is GI mucormycosis; it constitutes roughly 8% of all mucormycosis cases [4]. Symptoms of GI mucormycosis include abdominal pain, nausea/vomiting, and GI bleeding [5]. This case involves a 57-year-old man with uncontrolled diabetes admitted to the intensive care unit (ICU) for DKA due to a lack of adherence to his medications. The patient later developed anemia and was found to have GI mucormycosis and was successfully treated with antifungal therapy alone.

## Case presentation

A 57-year-old man with insulin-dependent type 2 diabetes mellitus and a history of prior admissions for DKA, who does not follow with an endocrinologist or primary care provider regularly, stating he goes years without seeing them, is noncompliant with his medications (hemoglobin A1c of 11.3 % on admission) and presented to the emergency department with respiratory distress. He was tachypneic, hypoxic, tachycardic, and hypotensive. The patient was immediately placed on bilevel positive airway pressure. A test of arterial blood gas with electrolytes showed an anion gap metabolic acidosis (Table 1). We subsequently intubated the patient and placed him on an insulin drip. The anion gap closed after a few days on an insulin drip and IV fluids. Blood glucose improved, allowing for a transition to long-acting insulin (Lantus), premeal insulin (Admelog), and an insulin sliding scale, which was transitioned to Admelog toward the end of admission due to intermittent episodes of hypoglycemia.

**Table 1:** Admission labs A test of arterial blood gas was done pCO_2_: partial pressure of carbon dioxide; pO_2_: partial pressure of carbon dioxide; HCO_3_: bicarbonate

Labs	Lab values	Normal
pH	7.29	7.35-7.45
pCO_2_ (mmHg)	28	35-45
pO_2_ (mmHg)	98	80-100
HCO_3_ (mEq/L)	14	22-26
Lactic acid (mmol/L)	10.41	0.5-2.2
Anion gap (mEq/L)	28	<12
Fasting blood glucose (mg/dL)	500	70-125

**Figure 1:**
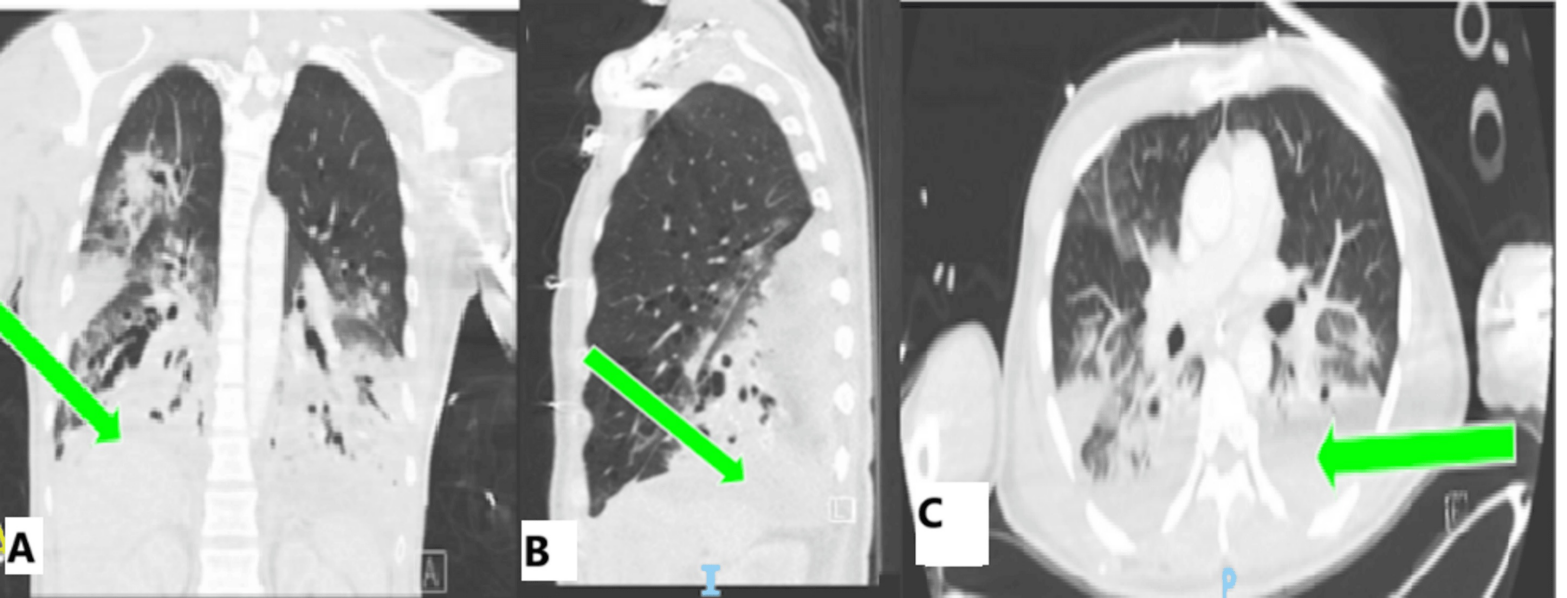
Computed tomography angiography of the chest and abdomen The computed tomography shows no signs of pulmonary embolism, small pleural effusions, no pneumothorax, and bilateral bronchiectasis. Also, there is extensive consolidation throughout all lobes, but the worst is in the lower lobes. Consolidation is shown in all planes: coronal (A), sagittal (B), and transverse (C)

The patient was leukopenic at 2.43 cells/uL (normal: 4.5-11). An infectious workup was done on the patient, and it was discovered that he had methicillin-sensitive *Staphylococcus aureus* (MSSA) bacteremia, MSSA in the endotracheal tube, and tested positive for enterovirus and rhinovirus. The patient was treated with Nafcillin, 2 g every four hours, for two weeks. The patient had increasing plateau pressures and was found to have acute respiratory distress syndrome with a partial pressure of oxygen/fraction of inspired oxygen ratio of 90 (normal: >400). He was placed in the prone position for the night. A CT angiogram of the chest and abdomen was performed, with no signs of pulmonary embolism, showing bilateral lower lobe consolidations and ground glass opacities concerning for pneumonia (Figure 1).

**Figure 2:**
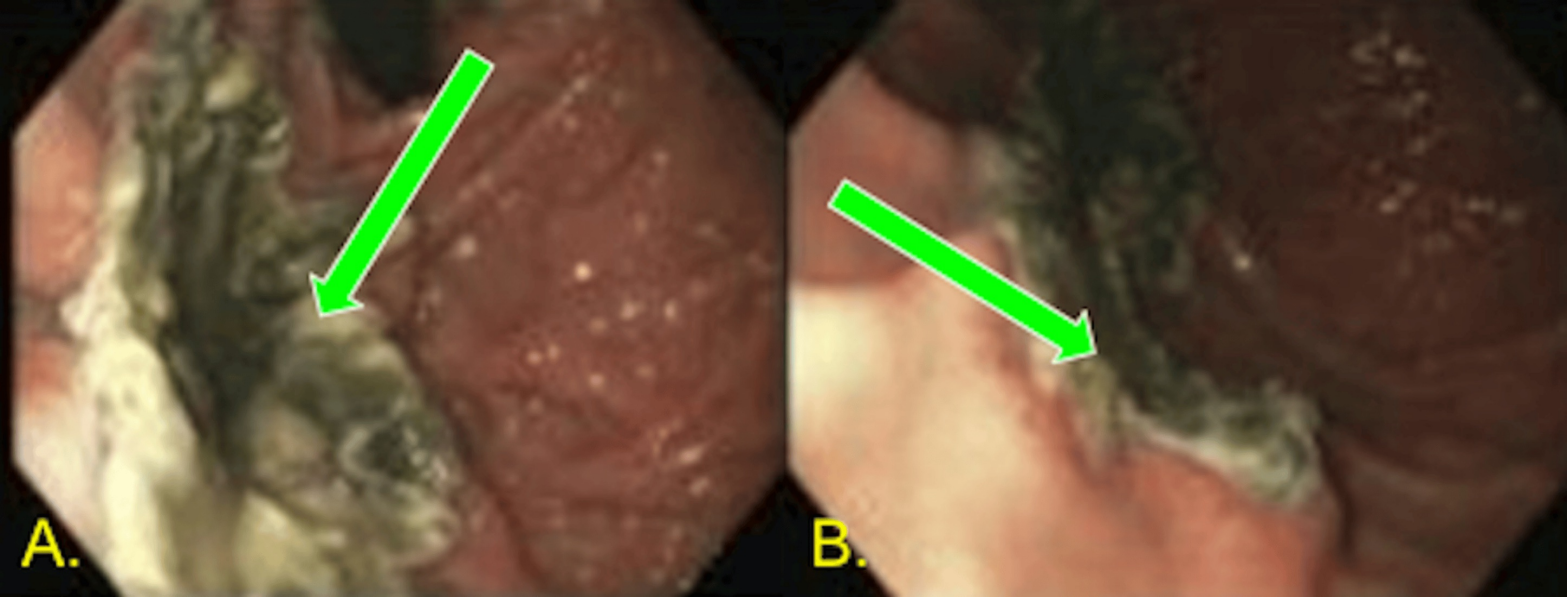
EGD with biopsies Images of endoscopy performed in the ICU. Images depict necrotic tissue in the fundus with heaped-up edges of ulcers underlying the necrotic tissue. (A) A more direct view. (B) A more oblique view EGD: esophagogastroduodenoscopy; ICU: intensive care unit

**Figure 3:**
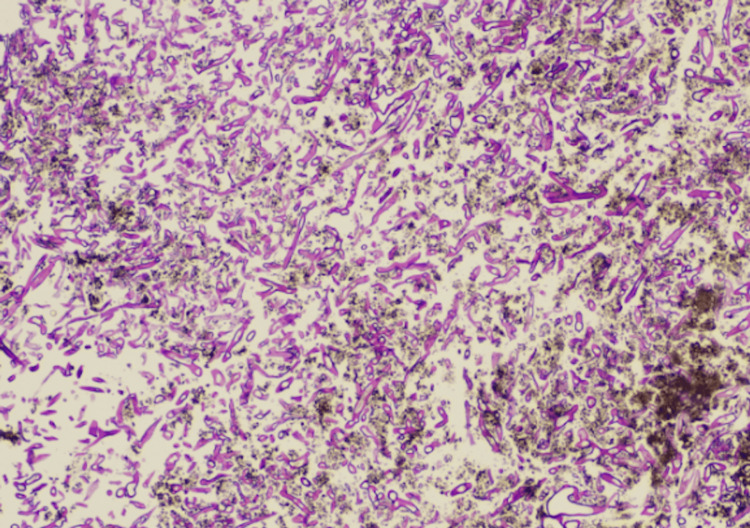
Histology slide of EGD biopsy 10× image depicting broad, nonseptate hyphae. The angle of branching is greater than in other organisms and usually approaches 90°. They are wider than Aspergillus species and branch irregularly. No background gastric mucosa was seen EGD: esophagogastroduodenoscopy

A few days after admission, the patient developed acute blood loss anemia requiring one unit of packed red blood cells. Repeat CT angiogram of the abdomen and pelvis showed pancolitis. The day after the repeat CT scan, the patient had a brisk upper GI bleed, prompting gastroenterology to perform esophagogastroduodenoscopy (EGD) (Figure 2), which showed large necrotic ulcers; biopsies were taken. The biopsies showed mucormycosis in the stomach (Figure 3). The patient was placed on amphotericin B 350 mg daily after three days from the procedure, as the biopsy needed confirmation by pathology. However, he was transitioned to isavuconazonium 372 mg every eight hours for two days, then 372 mg daily due to renal impairment after being on amphotericin. Creatinine began to increase after a week of therapy with amphotericin B. Creatinine peaked at 3.43 mg/dL, baseline Cr 0.80, and creatinine returned to normal two weeks later. Colonoscopy was not pursued as the bleeding was localized to the upper GI tract, and both GI polymerase chain reaction and stool studies showed no pathogens.

**Figure 4:**
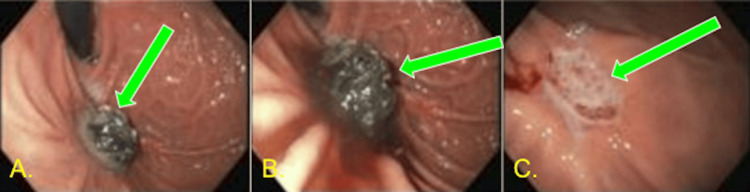
Repeat EGD after antifungal therapy (A,B) 1.5 cm fundal mass, consistent with a fungal ball, which had decreased in size. (C) A single-cratered, nonbleeding ulcer was found in the fundus, approximately 1.5 cm in diameter EGD: esophagogastroduodenoscopy

Repeat EGD was performed a few weeks after starting antifungals. There was a significant improvement in the follow-up EGD; the necrotic burden was reduced, and the ulcer bases were better visualized (Figure 4). Surgical debridement was considered; however, the patient refused. A few weeks after his stay in the ICU, the patient became septic again. Blood cultures were retaken, and extended-spectrum beta-lactamases *E. coli* bacteremia, which was sensitive to Meropenem, grew. The patient received Meropenem, 1 g every eight hours, for two weeks. The patient improved; repeat cultures 48 hours later had no growth. The patient was eventually weaned off pressors, decannulated from the tracheostomy tube, tolerated an oral diet, and was sent home after being on antifungals for two weeks. The patient followed up with gastroenterology and pulmonology two weeks after discharge. Upon discharge, the patient did not require supplemental oxygen and could walk and tolerate a normal diet. The patient remained on isavuconazonium 372 mg orally daily until his follow-up with infectious disease four weeks later. Given the severity of his gastric mucormycosis and its poor prognosis, his recovery was quite unexpected.

Differential diagnosis

The differential diagnosis for necrotic gastric tissue includes a range of infectious and noninfectious causes. Possible infections include fungal, bacterial, and viral infections, but noninfectious possibilities range from gastric cancer or lymphoma to ischemia, Crohn's disease, and medication-related injuries, such as those caused by nonsteroidal anti-inflammatory drugs. Other metabolic causes include diabetic gastropathy and uremic gastritis. A thorough evaluation with endoscopy, imaging, and lab testing was key in confirming the diagnosis of gastric mucormycosis.

Treatment

The patient was initially treated with liposomal amphotericin B 350 mg daily, the standard antifungal therapy for mucormycosis. However, after a week on the medication, renal impairment developed, so the treatment was changed to isavuconazonium, with a loading dose of 372 mg every eight hours for the first 48 hours, followed by a maintenance dose of 372 mg once daily. Other antifungal agents, e.g., posaconazole, were also considered but not used in this particular case.

Other supportive measures included pantoprazole twice daily for acid suppression, sucralfate 10 mL every six hours to aid mucosa healing, and enteral nutrition to meet caloric requirements.

His hospitalization was also marked by other treatments like anticoagulation with apixaban 5 mg twice daily for atrial fibrillation, prophylaxis of deep vein thrombosis, and individualized insulin therapy for glycemic control according to the standard inpatient protocol.

The patient was successfully discharged home after four weeks with the help of home care, supplemental oxygen (3 L nasal cannula), and the following medication regimen, including isavuconazonium 372 mg once daily, apixaban 5 mg twice daily, metformin 500 mg twice daily, pantoprazole 40 mg twice daily, sucralfate 10 mL every six hours, albuterol inhaler as needed every six hours, torsemide 20 mg once daily, and povidone iodine to the bilateral lower extremities twice daily.

## Discussion

Mucormycosis is a rare but severe fungal infection that primarily affects immunocompromised individuals, including those with diabetes mellitus, hematologic malignancies, or hematopoietic organ transplantation. In the United States, the incidence of mucormycosis has increased in the last few years, especially during the COVID-19 pandemic. The increasing incidence thus calls for early diagnosis and strict management to enhance the quality of life [6].

Mucormycosis should be clinically suspected and confirmed through histopathological examination of tissue and imaging. In this case, the endoscopic appearance of the necrotic tissue in the gastric fundus, with raised edges, confirmed by biopsy, was crucial in diagnosing. This agrees with previous studies, emphasizing the importance of early endoscopy and histological sampling for early infection detection and treatment initiation [7].

Mucormycosis is usually treated with a combination of surgery and antifungal agents. The source control is best achieved through surgical debridement and has been associated with lower mortality rates when combined with antifungal therapy. A systematic review and meta-analysis of the available literature, comprising 851 cases, revealed that combined surgical and antifungal treatment was associated with a lower odds of 90 days’ mortality compared to antifungal therapy alone [8]. However, another case report, combined with a literature review, found a slight difference between four of six cases treated with antifungals and five of ten cases treated with surgery and antifungals, which had a resolution of the disease [9]. The possible benefit of only using antifungals without surgery is on a case-by-case basis. The risk of surgery in these critically ill patients could explain it. Also, the possibility of manageable health conditions and the location of the infection have sufficient blood flow to allow for proper delivery of antifungals, making surgery unnecessary [10].

The primary agent of therapy for mucormycosis is liposomal amphotericin B. Liposomal amphotericin B is used due to its effectiveness and less nephrotoxicity compared to conventional amphotericin B. Posaconazole or isavuconazole can also be used in patients with renal dysfunction as alternative therapies. The patient in this report received liposomal amphotericin B and then isavuconazonium. Isavuconazonium is the prodrug of isavuconazole and has many advantages, including a broad spectrum of fungal activity, optimal pharmacokinetic properties, and a better safety profile in patients with renal insufficiency. Additionally, isavuconazonium is less likely to prolong the QT interval than azoles and has fewer drug interactions. All these features make isavuconazonium suitable for seriously ill patients [11-13].

Despite aggressive treatment, mucormycosis is a severe disease that can lead to serious complications and death. Previous studies have shown that the 90-day mortality rate is 35.3%, and the one-year mortality rate is almost 50% for the affected patients [4]. The poor prognostic factors are linked to the patient's age, leukemia, and CNS involvement. The prognosis is poor in critically ill patients, for example, those with hematologic malignancies, with ICU and hospital mortality rates of 77% and 88%, respectively. Furthermore, patients with mucormycosis have longer hospital stays, with a median of 17 days, and a high likelihood of readmission. Several case reports and case series have been published, describing the challenges and nuances of managing gastric mucormycosis. Reports vary from partial response to therapy and requirement for surgery to complete resolution with medical management only, with the overall outcome depending on the time of diagnosis and the patient's overall condition [14-16].

## Conclusions

Overall, this was a rare case of medically treated gastric mucormycosis. As highlighted above, gastric mucormycosis should be suspected clinically in critically ill patients with GI symptoms. Early recognition, appropriate management with antifungal agents, and getting surgery on board to weigh in on the case can improve outcomes. However, the decision to use only medical management instead of medical and surgical management for gastric mucormycosis cannot be made based on this case alone; it is a case-by-case basis, and further research must be done.
